# Effectiveness of a clinical practice guideline implementation strategy for patients with anxiety disorders in primary care: cluster randomized trial

**DOI:** 10.1186/1748-5908-6-123

**Published:** 2011-12-01

**Authors:** Eugenia Tello-Bernabé, Teresa Sanz-Cuesta, Isabel del Cura-González, María L de Santiago-Hernando, Montserrat Jurado-Sueiro, Mercedes Fernández-Girón, Francisca García-de Blas, Higinio Pensado-Freire, Francisco Góngora-Maldonado, María J de la Puente-Chamorro, Carmen Rodríguez-Pasamontes, Susana Martín-Iglesias

**Affiliations:** 1 Centro de Salud El Naranjo. Gerencia Atención Primaria. Servicio Madrileño de Salud. Spain; 2 Unidad Apoyo a la Investigación. Gerencia Atención Primaria, Servicio Madrileño de Salud, Spain; 3 Centro de Salud Castilla La Nueva. Gerencia Atención Primaria, Servicio Madrileño de Salud, Spain; 4 Centro de Salud Mª Ángeles López Gómez. Gerencia Atención Primaria, Servicio Madrileño de Salud, Spain; 5 Centro de Salud Mendiguchía Carriche. Gerencia Atención Primaria, Servicio Madrileño de Salud, Spain; 6 Centro de Salud Francia. Gerencia Atención Primaria, Servicio Madrileño de Salud, Spain; 7 Centro de Salud Humanes. Gerencia Atención Primaria, Servicio Madrileño de Salud, Spain; 8 Centro de Salud Santa Isabel. Gerencia Atención Primaria, Servicio Madrileño de Salud, Spain; 9 Dirección Asistencial Sur. Gerencia Atención Primaria, Servicio Madrileño de Salud, Spain

## Abstract

**Background:**

Anxiety is a common mental health problem seen in primary care. However, its management in clinical practice varies greatly. Clinical practice guidelines (CPGs) have the potential to reduce variations and improve the care received by patients by promoting interventions of proven benefit. However, uptake and adherence to their recommendations can be low.

**Method/design:**

This study involves a community based on cluster randomized trial in primary healthcare centres in the Madrid Region (Spain). The project aims to determine whether the use of implementation strategy (including training session, information, opinion leader, reminders, audit, and feed-back) of CPG for patients with anxiety disorders in primary care is more effective than usual diffusion.

The number of patients required is 296 (148 in each arm), all older than 18 years and diagnosed with generalized anxiety disorder, panic disorder, and panic attacks by the Diagnostic and Statistical Manual of Mental Disorders-IV (DSM-IV). They are chosen by consecutive sampling.

The main outcome variable is the change in two or more points into Goldberg anxiety scale at six and twelve months. Secondary outcome variables include quality of life (EuroQol 5D), and degree of compliance with the CPG recommendations on treatment, information, and referrals to mental health services. Main effectiveness will be analyzed by comparing the patients percentage improvement on the Goldberg scale between the intervention group and the control group. Logistic regression with random effects will be used to adjust for prognostic factors. Confounding factors or factors that might alter the effect recorded will be taken into account in this analysis.

**Discussion:**

There is a need to identify effective implementation strategies for CPG for the management of anxiety disorders present in primary care. Ensuring the appropriate uptake of guideline recommendations can reduce clinical variation and improve the care patients receive.

**Trial registration:**

ISRCTN: ISRCTN83365316

## Background

### Normal and pathological anxiety

Anxiety is a common mental health problem seen in primary care. Anxiety can be defined as an anticipation of future harm or misfortune, accompanied by a feeling of unpleasantness and/or somatic symptoms of tension. This feeling is normal in the face of certain day-to-day stressing situations. When it exceeds a certain intensity or overwhelms a person's adaptive capacity, anxiety becomes pathological. Anxiety disorders are a group of illnesses characterized by the presence of worry, excess of fear or dread, tension, or activation that causes a notable uneasiness or a clinically significant deterioration of an individual's activity [1].

The causes of anxiety disorders are not entirely known, but biological factors, as well as environmental and psychosocial ones, are involved [2-4]. Some authors say that it is the interaction of multiple determining factors that favours the appearance of these anxiety disorders [5]. In addition, co-morbidity with other mental disorders, such as mood disorders, is very common [3-6].

The proper diagnosis, treatment, and, in those cases where necessary, appropriate referral of anxiety disorders to mental health services, is fundamental for effective clinical management [7,8].

### Anxiety as a health problem

The prevalence of anxiety disorders varies depending on different epidemiological studies, with prevalence-year and prevalence-life being set at between 10.6% and 16.6% [9]. Data for Spain from the European Study of the Epidemiology of Mental Disorders (ESEMeD) estimate a prevalence-year of 5.1% [10,11]. Mental disorders represent a burden for individuals, as well as for families and the community. In the case of the community of Madrid, neuropsychiatric illnesses are confirmed as the first cause of adjusted years of life for incapacity [12].

According to the World Health Organization's report on mental health, 20% of all patients attended by primary care professionals suffer one or more mental disorders. This report recommends that management and treatment be performed as far as possible in primary care so as to facilitate access to services for the greatest number of people possible [13].

### Clinical practice guidelines (CPGs) as a tool for the management of anxiety disorders in primary care

The diagnostic and therapeutic approach to these disorders varies widely. Primary care professionals have different grades of training in the area of mental health, in psychiatric interview skills, as well as in psychological intervention techniques [14-16].

A study in the United States between 1985 and 1998 described an increase in the use of psychopharmaceutical medication in primary care visits (38.7% compared with 54.6%) while, at the same time, it showed a constant drop in the use of psychological interventions (6.8% to 2%) in the treatment of anxiety disorders [17].

CPGs are a good tool to reduce this variability and improve evidence informed in the clinical decision-making. In Spain, the Ministry of Health and Social Policy has put into effect the 'Guiasalud' project [18] to draft CPGs. In the framework of this project, the *Clinical Practice Guideline for the Approach to Anxiety Disorders in Primary Care *has been drafted in coordination with the Technologies Assessment Unit of the Agencia Lain Entralgo [19].

The scope of the CPG covers the diagnosis and management of adult patients with generalized anxiety disorders (GAD) and panic disorder (PD), with or without agoraphobia, in the context of primary care.

Two aspects of this CPG development are worth noting. The first is the involvement of patients with anxiety disorders in all phases of the development process. The second is that the CPG includes interventions that can be carried out by the various professionals working in primary care. These interventions are carried out at the primary healthcare centers, mainly by nursing and/or social workers [19]. Likewise, it is worth noting the low utilization of psychological interventions of proven effectiveness [20]. Our group has carried out a study that focuses on the evaluation of the effectiveness of the intervention groups directed by nurses in patients with anxiety in primary care [21].

The Goldberg Anxiety and Depression Scale (GADS) has been included in the guideline to evaluate the changes obtained by the various interventions and as a way to provide key questions to guide the clinical interview. This scale has been chosen because it is brief and easy to manage and interpret [22-24]. The Spanish version has demonstrated its reliability and validity within the ambit of primary care, and it has the right sensitivity (83.1%), specificity (81.8%), and positive predictive value (95.3%) [25].

### Strategies to implement CPGs

CPGs will only be useful for professionals and patients if their recommendations are incorporated to regular clinical practice. Achieving this is a complex process in which several factors play a role [26,27].

To increase the use of the guideline, its distribution and implementation strategy must be planned very carefully. As a first step, the identification of the barriers and facilitating factors, adapting all strategies to the setting in which the CPGs will be used, is fundamental [28-30].

Several CPGs implementation strategies have demonstrated their effectiveness. Some authors group these interventions depending on whom they are addressed to: physicians and patients, communities or the general population, or health centres and/or health systems [31].

The Cochrane EPOC (Effective Practice and Organization of Care) group proposes to classify interventions into four categories: professional interventions (such as distribution of educational materials or educational meetings); financial interventions (such as fee-for-service or prospective payment); organisational interventions (such as creation of clinical multidisciplinary teams) and regulatory interventions (any intervention that aims to change health services delivery or costs by regulation or law) [32].

The Grimshaw [33]*et al*. review evaluates the effectiveness and cost of different clinical practice guideline dissemination-implementation organizational strategies. This study takes into account the impact on professionals as well as on patients. It includes 235 studies (between 1966 and 1998) and 309 comparisons. Among the results, it is worth noting that most implementation strategies improved adherence to CPGs. Simple strategies, such as reminders, increase adherence by 14.1%. The distribution of educational materials improves this by 8.1%. Educational programmes, almost always as components of more complex interventions, improved practice by 6%, and audits and feedback by 7%. The complexity of the strategies and the mixture of interventions did not improve results. In their conclusions, it is noted that there is little evidence about which strategies can be more effective in each situation. The authors consider that there is a need to develop and validate theoretical models for behavioural change, as well as to investigate the efficiency of the strategies in the presence of different barriers and factors that can modify effectiveness.

We consider it fundamental to put into effect organizational strategies that will enable us to use the guideline and apply its recommendations, as well as to measure and evaluate the impact that implementation of the CPGs will have on professionals modifying their practices, and improving outcomes for patients [34,35].

There are two reasons for using cluster randomised trials design: to evaluate the group effect of an intervention; and to avoid 'contamination' across interventions when trial participants are managed within the same setting.

### Main objective

The aim of the present work is to determine whether the use of a CPG implementation strategy (including training session, information, opinion leader, reminders, audit, and feed-back) for patients with anxiety disorders in primary care is more effective than usual diffusion in improving the score of the Goldberg anxiety scale at six and twelve months.

### Secondary objectives

1. Evaluate the effectiveness of a CPG implementation strategy for the management of patients with anxiety disorders compared with the regular strategy, measured as the degree of suitability of the treatments (psychological, pharmacological, *et al*.) received by patients.

2. Evaluate the effectiveness of a CPG implementation strategy for the management of patients with anxiety disorders compared with the regular strategy, measured as the percentage of patients who have received the information proposed in the guideline (oral or written) about their disorder.

3. Evaluate the effectiveness of a CPG implementation strategy for the management of patients with anxiety disorders compared with the regular strategy, measured as the degree of suitability of referral to mental health services, based criteria established in the guideline.

4. Describe the professionals' opinion about the usefulness of the CPG.

5. Evaluate the effectiveness of a CPG implementation strategy for the management of patients with anxiety disorders compared with the regular strategy measured as a change in the patient's quality of life.

## Methods/design

### Design of the study

This study is a two-year community, parallel clinical trial, randomised by clusters, that compares two different CPG implementation strategies. The intervention will be made on health professionals (physicians, nurses, and social workers) of primary healthcare centres (PHCC) in the region of Madrid, Spain. The randomization units will be PHCCs (clusters). The units of analysis are patients of health professionals (individual level) of the PHCCs participating in the study (as a control or intervention) according to the CONSORT checklist (additional file 1).

### Subjects of the study

Subjects older than 18 years of age, who visit primary care because of symptoms compatible with anxiety disorders at PHCCs in the region of Madrid, and those who have been diagnosed with anxiety disorders according to Statistical Manual of Mental Disorders-IV (DSM-IV) criteria [1].

### Inclusion criteria

Agree to participate in the study and written informed consent.

### Exclusion criteria

1. Subjects diagnosed with anxiety for post-traumatic stress, acute stress disorders, anxiety disorders induced by substances, organic anxiety disorders.

2. Subjects who cannot read or understand Spanish.

3. Subjects who will not reside in the basic area in the year following their inclusion in the study.

4. Immobilized or institutionalized patients.

### Sample size

#### Method of calculation

For an alpha of 0,05, a power of 80% and in order to detect a decrease in the Goldberg Scale (two or more points) of 20% in the intervention group, the overall simple size required 206 patients (103 in each arm of the study). Because randomisation was by cluster, the sample size had to be larger than if simple randomisation had been performed, in order to take into account the design effect (DE). The DE was calculated as follows: DE = 1 + (n_c _- 1) * ICC (where n_c _is the mean number of individuals in the cluster, and ICC the intracluster correlation coefficient) [36]. The ICC in the present work was deemed to be 0.01. The mean cluster size was assumed to be 20 patients. Given these assumptions, and expecting a 20% loss rate, the final sample size required was 296 patients (148 in each arm).

### Randomisation

#### Unit of allocation

Allocation will be performed by clusters, the PHCC being the randomisation unit. This will minimise possible contamination effects between professionals.

### Sequence generation

The 16 PHCCs will be assigned to the intervention or the control group following a simple, computer-generated random sequence (EPIDAT 3.1).

### Concealment of allocation

Randomization will be performed centrally by a researcher not involved in the study, and who was blind to the identity of the PHCCs.

Consecutive patients will be chosen to minimise the risk of bias in their selection. During consultations, patients will be informed about the study and asked whether they would like to take part in it. Those who accept will be asked to complete a signed consent form. Checks will be made to ensure they met all inclusion criteria, but no exclusion criterion.

### Masking

In the study, the patients, the professionals implementing the interventions, and those assessing the outcomes will not be blinded to the assignment group. However, analysis data will be performed by independent professionals blinded to the assignment group.

### Intervention (see Table 1)

**Table 1:** Interventions to professionals in both groups

Step 1	Centralized clinical session for the 16 PHCCs in which the GPC will be presented. Website 'Guiasalud'. Invitation to participate.	
Step 2	Primary Healthcare Centre RANDOMIZATION	

Step 3	Intervention group	Control group
	
	IMPLEMENTATION STRATEGIES: - Information and training about diagnosis and therapeutic plan - Election of opinion leader - Information and training structured to create anxiety disorder groups	Regular care

Step 4 (after visit 2)	IMPLEMENTATION STRATEGIES: - Audit and feedback - Reminders	Regular care

Step 5 (after visit 3)	IMPLEMENTATION STRATEGIES: - Audit and feedback - Reminders	Regular care

#### Control group

1. The CPGs will be searchable on the intranet corporative system.

2. Centralized area clinical session with a duration of 60 minutes, to which each one of the professionals from each of the 16 health centres will be invited, and where the CPGs will be presented.

#### Intervention group

The intervention, through the different strategies, has been designed to allow professionals to understand the guideline thoroughly, and also to implement its recommendations.

1. The CPG will be searchable on the intranet corporative system.

2. Centralized area clinical session with a duration of 60 minutes, to which each one of the professionals from each of the 16 PHCCs will be invited and where the CPG will be presented.

3. Information: Presentation of CPG in the PHCCs.

4. Training: two sessions with a duration of 60 minutes, imparted at PHCCs by the researchers.

5. Information and training to physicians, nurses, and social workers with the following contents:

a. Information and diagnosis: This would focus on the questions in the guideline that approach the definition of anxiety and its differentiation as a symptom and as a disorder. It would approach the semi-structured interview at the visit with an active methodology (roll-playing, *et al*.).

b. Pharmacological treatment and algorithms.

c. Non-pharmacological treatment: Group techniques (see table 2), bibliotherapy and medicinal herbs.

**Table 2:** Group intervention in anxiety

First session	
	Presentation of the group General introduction to program Educational component:
	• What is the response to anxiety?
	• Why does it happen in daily life?
	• Is recovery possible?
	• What is the recovery process?
	The role of lifestyle in the response to anxiety (Part 1):
	• What do we do to take care of ourselves?
	• How lifestyle influences.
	• Introduction to relaxation and breathing.

**Second session**	

	Introduction, we clarify doubts. The interpretation of body sensations. What is an anxiety crisis? Why does it appear? Understand the role thoughts-behaviour-emotions play in the maintenance of the problem. The role of lifestyle in the response to anxiety (Part 2):
	• Physical exercise
	• We learn to breath (control of breathing)
	• Relaxation training

**Third session**	

	We review relaxation training execution and follow up with respect to exercise. The role of lifestyle in the response to anxiety (Part 3):
	• Eating/consumption toxics/stimulants
	• Control of breathing
	• Relaxation training

**Fourth session**	

	We review relaxation training execution and follow up with respect to exercise. The role of lifestyle in the response to anxiety (Part 4):
	• Pace activity/rest (sleep)
	• Control of breathing
	• Relaxation training

**Fifth session**	

	We review control of breathing and relaxation training execution.

**Sixth session**	

	We review control of breathing and relaxation training execution. Using what we learned to face future changes: integrating vision to remember what we learned. Depending on availability, the group intervention can be extended to eight sessions.

2. Opinion head/leader: a professional designated at each centre (physician, nurse or social worker).

3. Structured training: Training of nurses and social workers to carry out anxiety disorder groups of six to eight sessions.

4. Reminders: Reminders will be sent in the form of letters or electronic flashes or systems of alert about publications.

5. Audit: The results will be audited and will be returned to the PHCCs through the local leader.

### Patient variables (individual level)

#### Variables to measure health outcomes

1. Degree of anxiety, measured by the Goldberg anxiety subscale. The decrease in the score by two or more points compared with baseline score will be evaluated at six and twelve months.

2. Quality of life measured by means of the EuroQol 5D questionnaire. The quality of life will be evaluated at baseline, six months and twelve months.

### Clinical variables recorded to check the degree of suitability of the guideline recommendations

Previous episodes and treatment, diagnosis and current therapeutic plan, and reason for referral to mental health.

### Sociodemographic variables recorded

Age, gender, nationality, time of residence in Spain, and employment status.

### Variables recorded for family physicians (cluster level)

Age, gender, years of professional activity, training in interview skills, training in psychological intervention techniques, average number of patients attended in three months, and evaluation about usefulness of the guide and modification of its clinical practice.

### Data collection method

#### Professionals

Data will be collected by telephone interview by a member of the research team before randomisation. All data will be collected except the number of patients visited, which will be taken from the health information system. Opinion will be collected at the end of the study.

### Patients

**Figure 1:**
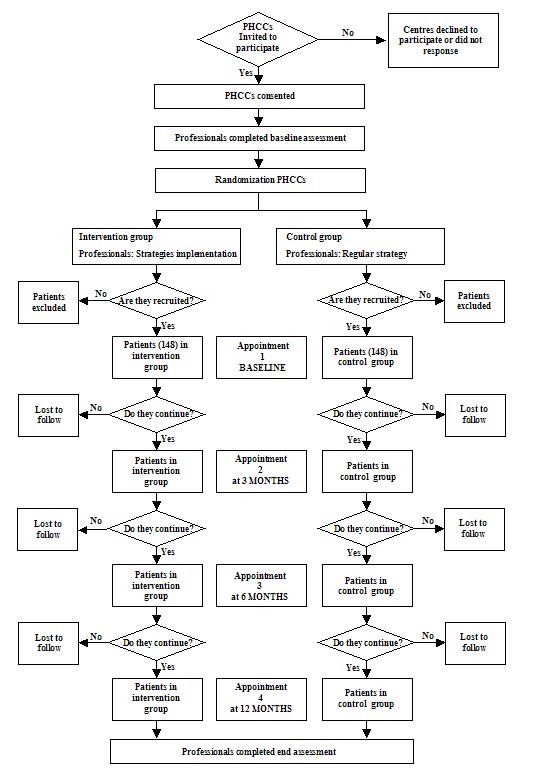
**Flowchart**.

The clinical interview will be used and the data will be recorded in an electronic data collection log especially designed for this study. The different variables will be collected over four follow-up visits: baseline, three, six, and twelve months. The Figure 1 shows the flowchart of the study.

### Statistical analysis

This will be based on the principle of intention to treat. The use of the design by clusters chosen will be taken into account in all phases of data analysis, especially in the calculation of the confidence intervals (CI) of the estimates and in the hypothesis tests:

1. Descriptive analysis of each variable with its corresponding CI at 95%. Tests for normality. Description of losses and quitting.

2. Comparison of the group at the beginning of the trial with regards to response variables, descriptive variables, and prediction factors. Bivariate statistical tests will be used suitable to the type of variable (qualitative or quantitative).

3. Analysis of primary outcome. There will be a comparison of the percentage of patients with a reduction of two or more points on the Goldberg anxiety subscale at six and twelve months from the intervention in both groups by the chi-squared test, and the confidence interval of the difference will be calculated. Confounding variables will be controlled with multivariate analysis based on logistic regression with random effects.

4. Analysis of secondary outcome. For each of the secondary response variables the results of the variables will be compared based on the group assigned by calculating the difference in proportions of each variable.

### Ethical considerations

The study protocol was approved by the Research Ethics Committee on 29 April 2010 and met all good clinical practice demands right. This study has been funded by the Spanish Ministry of Science and Innovation via the Instituto de Salud Carlos III (PI09/90304). The trial was registered with Current Controlled Trials, number ISRCTN83365316.

[http://www.controlled-trials.com/ISRCTN83365316]

## Discussion

The proposed implementation strategy should have impact at four levels: increasing the knowledge of clinical physicians and patients; changing attitudes; changing the habits and behaviour of professionals in their clinical practice, while taking into account the patient's preferences, as well as administrative and economic influences; and modifying results, which means improving the quality of care and, finally, the health of the population by following CPG recommendations [37,38]. In this study, we will focus on checking the degree of suitability of the guideline recommendations (in terms of treatment, referral to mental health services, and information to the patient) in the clinical practice. We will also check if health outcomes (anxiety and quality of life) are improving.

If the intervention proposed is effective, it could be generalized to other common problems present in primary care. The findings of the study can also inform a future updates of the guideline. The 'Guiasalud' project includes a methodological manual for GPCs implementation [39]; the study outcomes may serve, moreover, to evaluate this manual.

In addition, our project seeks to enhance the importance of multidisciplinary teams working in the design, implantation and evaluation of CPGs.

As for the limitations of this study, the design selected for this project is the best possible given the intervention, while seeking to avoid possible contamination. Although the number of clusters is enough for the randomization to balance the potentially confounding factors among themselves, the following must be taken into account:

1. Given the nature of the study, the intervention cannot be masked.

2. There may be a classification bias, given the difficulty professionals have for making specific diagnoses, but this should be minimized by the information contained in the guideline.

3. Patients will be enrolled in the study by their own professionals. This increases variability in the approach, which will be minimized, on the one hand, by the training of all participating professionals and, on the other hand, by the protocolization of the research and the implementation of the computer application of the data collection log in the computer programme. Moreover, the fact that the professionals themselves enrol patients will increase applicability in regular practice.

4. Although losses are contemplated in the follow-up, these are reduced in primary care because of the proximity of users to the system.

5. There may be a selection bias, because the selection and inclusion of patients will take place after randomisation of clusters. In order to be able to evaluate this bias, the information of those persons who decline to participate for each intervention group will be collected and compared.

In summary, the main contribution of our project is that it seeks to identify an effective strategy for implementing a CPG for the management of a common disorder present in primary care. Ensuring the appropriate uptake of guideline recommendations can reduce clinical variation and improve the care patients receive.

## Competing interests

The authors declare that they have no competing interests.

## Authors' contributions

ETB conceived the study. ETB, ICG and TSC participated in the design and coordination. MLSH, MJPC, MFG, FGM, MJS, SMI, HPF, and CRP collaborated in the methodology, the design of the intervention, and the bibliographical search. ETB, ICG, TSC, and FGB wrote the manuscript. All the authors critically evaluated the content of this article until the final version was approved.

## Supplementary Material

Additional file 1 **CONSORT checklist**. Checklist of items to include when reporting a cluster randomised trial. Click here for file
